# Prevalence of Postoperative Atrial Fibrillation Following Off-Pump Coronary Artery Bypass Graft Surgery in Elderly Patients

**DOI:** 10.7759/cureus.34499

**Published:** 2023-02-01

**Authors:** Ashok Kumar, Redoy Ranjan, Asit Baran Adhikary

**Affiliations:** 1 Department of Cardiac Surgery, Bangabandhu Sheikh Mujib Medical University, Dhaka, BGD

**Keywords:** duration of the hospital stays, icu stays, off-pump coronary artery bypass graft surgery, elderly, postoperative atrial fibrillation

## Abstract

Background

Atrial fibrillation (AF) is one of the frequent complications following coronary artery bypass surgery. Postoperative atrial fibrillation (POAF) can lead to thromboembolic events and prolong hospital stays. We aimed to determine the prevalence of POAF following off-pump coronary artery bypass surgery (OPCAB) in the elderly population.

Materials and Methods

This cross-sectional study was carried out between May 2018 to April 2020. Elderly patients (age ≥65 years) admitted for isolated elective OPCAB were eligible for the study. A total of 60 elderly patients were evaluated based on the preoperative and intraoperative risk factors and postoperative outcomes during the hospital stay.

Results

The mean age was 67.83±4.06 years, and the prevalence of POAF in elderly adults was 48.3%. The mean number of grafts was 3.20±0.73, and ICU stays at 3.43±1.61 days. The mean duration of the hospital stays was 10.03±2.12 days. Although stroke developed in 1.7% of post-CABG patients, no mortality was observed postoperatively.

Conclusion* *

POAF is one of the commonly encountered complications following OPCAB. Though OPCAB is a superior revascularization procedure, preoperative planning and attention are especially needed in the elderly to reduce the prevalence of POAF.

## Introduction

Atrial fibrillation (AF) presents a difficult clinical challenge and accounts for about one-third of diagnosed arrhythmia. About 6% of people over 65 years have a history of AF [1]. The annual incidence of AF in Asian countries is 5.38/1,000 people [2]. The pathophysiology of postoperative atrial fibrillation (POAF) focuses on identifying risk factors for initiating the arrhythmia. Re-entry involving one or more circuits and enhanced automaticity in one or several rapidly depolarizing foci are the theories concerning the mechanism of AF [3]. Coronary artery bypass grafting (CABG) is indicated in severe coronary artery disease (CAD) and can be revascularized by conventional on-pump CABG or off-pump CABG (OPCAB). OPCAB is associated with lower perioperative complications and hospital mortality with respect to conventional CABG [4].

Rhythm disturbances in the postoperative period depend on electrolyte shifts related to revascularization, temporary ischemia, perioperative trauma, epicardial inflammatory reaction, and transient increase of sympathetic activity [5]. POAF occurs in 20% to 40% of patients after CABG [1]. In patients undergoing OPCAB, the incidence of POAF is 19.4% [6]. POAF usually occurs within the first three days after CABG, although it may develop later. More than 90% of patients have no prior history of atrial arrhythmia and are in normal sinus rhythm within 6 to 8 weeks after. The cause of POAF includes age and its accompanying degenerative changes in the atrial myocardium [1]. The left atrial (LA) enlargement and left ventricular diastolic dysfunction resulting from aging increase the susceptibility of the elderly to AF [7]. The advancement of age is an independent predictor of POAF patients undergoing CABG [8]. Increased preoperative plasma BNP level independently predicts POAF [9]. In women, the incidence of AF following CABG is lower [10].

Systemic arterial hypertension, decreased left ventricular function, chronic obstructive pulmonary disease (COPD), chronic renal failure, diabetes, and withdrawal of β-blockers are also responsible for POAF [1]. Smoking, LDL, statins, LA diameter, and left ventricular ejection fraction (LVEF) are significant risk factors for POAF preoperatively [11]. Preoperative HbA1c independently predicts the occurrence of AF after isolated OPCAB [12]. AF is associated with hyperthyroidism and alcohol consumption [13]. Predictors of POAF include the number of vessels bypassed, location of coronary anastomosis (diagonal or posterior descending artery), and net fluid balance on an operative day [14].

Operative trauma from surgical dissection and manipulation, local inflammation with or without pericarditis, and elevations in atrial pressure from postoperative ventricular stunning are responsible for the prevalence of POAF. Chemical stimulation with catecholamines and other inotropic agents plays a role in developing POAF. Reflex sympathetic activation from volume loss, parasympathetic activation, anemia or pain, fever from atelectasis or infection, and hypoglycemia incurred during the operation also predispose to developing POAF [15]. Transient regional ischemia of the myocardium due to surgical methods applied in OPCAB shows the influence of the technique on the incidence of POAF [5].

This study focused on evaluating the occurrence and consequences of POAF following off-pump coronary artery bypass surgery (OPCAB) in the elderly population in Bangladesh.

## Materials and methods

This cross-sectional study was performed in the Department of Cardiac Surgery, Bangabandhu Sheikh Mujib Medical University, Shahbagh, Dhaka, Bangladesh, from May 2018 to April 2020. The patients aged 65 years and above were considered elderly as per the definition of ‘elderly’ by Geriatrics and Gerontology International [16]. The purposive sampling method was applied as the sampling technique. The study population was 60.

During this study period, every patient admitted for CABG was approached, and data were collected by face-to-face interview and a semi-structured questionnaire. Inclusion criteria were patients admitted for isolated elective OPCAB. Exclusion criteria were pre-existing AF and other arrhythmias, taking antiarrhythmic drugs except for beta blockers, pre-existing thyroid disorders, congestive cardiac failure, concomitant other cardiac procedure, acute coronary syndrome within two months, emergency OPCAB, hepatic impairment, chronic kidney disease stage 3b-5, previous stroke, significant carotid artery disease and intraoperative conversion to on-pump CABG. We used both primary and secondary data sources for this study. The primary sources were collected via face-to-face interviews and semi-structured questionnaires, and the secondary sources were obtained through clinical observations and laboratory workup.

Potential preoperative risk factors analyzed in this study were age, sex, BMI, smoking, alcohol consumption, shortness of breath, chest pain, hypertension, diabetes mellitus, dyslipidemia, COPD, and history of old MI. In addition, LA diameter, LVEF, number of diseased vessels, double vessel disease (DVD), triple vessel disease (TVD), left main stem involvement, right coronary artery (RCA) total/near total occlusion, in-stent restenosis, and laboratory workup were analyzed. Intraoperative characteristics included duration of surgery, number of grafts, and coronary endarterectomy. Postoperative characteristics were laboratory workup, POAF, POAF episodes duration, restoration of sinus rhythm from POAF, duration of ICU stays, duration of hospital stays, stroke, and mortality. The patient who fulfilled the inclusion criteria, other than the exclusion criteria, and was willing to enroll in this study after giving the proper consent underwent elective isolated OPCAB.

Surgical technique and POAF monitoring

Anesthetic induction and maintenance were done following standard protocol. All patients were operated through a median sternotomy approach. Left internal mammary artery (LIMA) and great saphenous vein (GSV) were harvested. Activated clotting time (ACT) was kept at>350 seconds just before completing the LIMA harvest. Proximal anastomoses were performed using a side-biting clamp following the parachute technique. The heel of the proximal anastomosis was at a 3 or 4 o'clock position for the left side and a 6 o'clock position for the right side. Distal grafting was done LIMA to LAD first for left main stem involvement. Then depending on the severity of the lesion, we performed distal grafting in either circumflex or RCA territory. Target coronary arteries were stabilized using the tissue stabilizing system, and appropriate-sized intracoronary shunts were used in all cases to maintain distal perfusion and to achieve a bloodless operative field. After completion of the surgery, all patients were shifted to the intensive care unit (ICU), and mechanical ventilation was continued until the patient met the standard extubation criteria. In ICU, all patients were monitored for heart rate and rhythm with continuous ECG monitoring. ECG monitoring was continued throughout the postoperative period during the hospital stay. In addition, a 12 lead ECG was performed whenever POAF was suspected by continuous monitoring.

Data analysis

Statistical analyses were done by using IBM Corp. Released 2017. IBM SPSS Statistics for Windows, Version 25.0. Armonk, NY: IBM Corp. For continuous variables, data were expressed as Mean±SD, and for categorical variables, as frequency and percentages.

Ethical considerations

Ethical clearance was taken from the concerned departmental, academic, and technical committees and the Institutional Review Board (IRB), Bangabandhu Sheikh Mujib Medical University, Shahbagh, Dhaka, Bangladesh. From all the respondents of this study, informed written consent was taken. The validity of the observations and findings was confirmed by the quality assurance system. The reliability of the data was ensured to validate that it was derived correctly from the raw data. Quality control measures were taken in every stage of data handling to ensure consistency and appropriate processing of data.

## Results

About 60 elderly adult patients underwent isolated elective OPCAB. The mean age was 67.83±4.06 years, and 86.6% were male. The mean BMI was 25.59±3.01 Kg/m2. The most common risk factors associated with CAD were HTN (81.7%), diabetes (46.7%), and smoking (45%). About 91.7% of the study population has a history of old MI. A higher cardiothoracic ratio (CTR) was present in 30% of the subjects (Table 1).

**Table 1:** Demographic, anthropometric, and clinical characteristics BMI: Body Mass index; COPD: Chronic obstructive pulmonary disease; MI: Myocardial infarction: BNP: B-type natriuretic peptide.

Variables	n = 60 f (%)	Mean±SD
Age (years)		67.83±4.06
Sex		
Male	52 (86.6%)	
Female	08(13.3%)	
BMI(Kg/m^2^)		25.59±3.01
Smoking	27(45%)	
Alcohol consumption	03(5%)	
Shortness of breath	11(18.3%)	
Chest pain	55(91.7%)	
Hypertension	49(81.7%)	
Diabetes Mellitus	28(46.7%)	
Dyslipidemia	46(76.6%)	
COPD	05(8.3%)	
History of old MI	55(91.7%)	
Higher cardiothoracic ratio (CTR)	18(30%)	
Serum creatinine (mg/dl)		1.29±0.23
Plasma BNP (pg/ml)		150.24±81.34

The mean LA diameter was 43.61±6.48 mm, and the mean LVEF was 41.42±7.29 %. The mean number of diseased vessels was 2.77±0.42. DVD and TVD were found in 23.30% and 76.70% of patients, respectively. This study also observed the presence of left main stem involvement and RCA total/near total occlusion in the elderly was 45% and 43.33%, respectively. In-stent restenosis was observed in 16.7% of patients (Table 2).

**Table 2:** Preoperative echocardiographic and angiographic characteristics LA: Left atrial; LVEF: Left ventricular ejection fraction; DVD: Double vessel disease; TVD: Triple vessel disease: RCA: Right coronary artery.

Variables	Mean±SD	n = 60 f (%)
LA diameter (mm)	43.61±6.48	
LVEF (%)	41.42±7.29	
Number of diseased vessel	2.77±0.42	
DVD		14(23.30%)
TVD		46(76.70%)
Left main stem involvement		27(45%)
RCA total/near total occlusion		26(43.33%)
In-stent restenosis		10(16.7%)

The mean duration of surgery was 4.79±0.65 hours, and 3.20±0.73 grafts were performed per patient. Endarterectomy was done in 33.3% population. About 45% of endarterectomy was done in the left anterior descending artery (LAD) territory (Table 3).

**Table 3:** Intraoperative characteristics Note: Territory of coronary endarterectomy (n=20)

Variables	Mean±SD	n = 60 f (%)	n = 20 f (%)
Duration of surgery (hours)	4.79±0.65		
Number of grafts	3.20±0.73		
Coronary endarterectomy		20(33.3%)	
Territory of coronary endarterectomy			
Left anterior descending artery (LAD)			09(45%)
Obtuse marginal artery (OM)			01(5%)
Diagonal artery			05(25%)
Right coronary artery (RCA)			03(15%)
Posterior descending artery (PDA)			02(10%)
in double vessel			02(10%)

Postoperative serum electrolyte levels are shown in Table 4.

**Table 4:** Postoperative serum electrolytes level (n=60) POD: Postoperative day.

Postoperative serum electrolytes (mmol/L)	Mean±SD
Serum Sodium (Na^+^) level (mmol/L)	
POD 0	138.02±3.63
POD 1	136.80±3.23
POD 2	136.26±3.42
POD 3	136.06±2.98
Serum Potassium (K^+^ ) level (mmol/L)	
POD 0	4.05±0.38
POD 1	4.06±0.42
POD 2	4.04±0.36
POD 3	4.03±0.20
Serum Calcium (Ca^++^) level (mmol/L)	
POD 0	1.16±0.03
POD 1	1.14±0.04
POD 2	1.15±0.06
POD 3	1.17±0.04

POAF developed in 48.3% of the study population, out of which 79.31% of subjects returned to sinus rhythm. The mean duration of ICU stays was 3.43±1.61 days, and the mean duration of hospital stay was 10.03±2.12 days (Table 5).

**Table 5:** Postoperative outcome variables Note: POAF episodes duration (n=29) POAF: Postoperative atrial fibrillation; POD: Postoperative day; ICU: Intensive care unit.

Variables	n = 60 f (%)	n = 29 f (%)	Mean±SD
Hemoglobin (gm/dl) in POD 1			12.03±1.33
Plasma BNP (pmol/L) in POD 1			198.31±97.64
POAF	29 (48.3%)		
POAF episodes duration (<24 hours)		21(72.41%)	
POAF episodes duration (24-48 hours)		01(3.44%)	
POAF episodes duration (2-7 days)		01(3.44%)	
POAF episodes duration (>7 days)		06(20.68%)	
Restoration of sinus rhythm from POAF (≤7 POD)		23(79.31%)	
Duration of ICU stay (days)			3.43±1.61
Duration of Hospital stay (days)			10.03±2.12
Stroke	01(1.7%)		
Mortality	0(0%)		

## Discussion

Postoperative atrial fibrillation (POAF) contributes significantly to postoperative morbidity following coronary artery bypass graft surgery. AF is a disorder of cardiac rhythm characterized by rapid, irregular, disorganized atrial impulses and ineffective atrial contractions [1]. Advanced age is an independent predictor of POAF patients undergoing CABG [8,17]. The mean age of the elderly was 67.83±4.06 years. Among the study population, male was predominant, which reflects the male predominance for CAD [18,19]. BMI is not associated with POAF [20]. Hypertension is related to the development of POAF [21]. We found that most of our patients are hypertensive, which indicates a possible association with POAF. Our study observed a higher prevalence of old MI in the elderly. The observations of our study are similar to previous studies [1,22].

Tavakol and colleagues found plasma BNP values were insignificantly higher preoperatively and postoperatively in patients with AF [23]. We observed higher mean plasma BNP levels in the preoperative period, which might be associated with POAF, although other conditions may cause an elevation of plasma BNP. Xu and colleagues reported that increased LA size is related to POAF [11]. We found increased mean LA size among our study populations, and these findings have also been confirmed [7]. RCA occlusion has no significant relationship with POAF [24]. But our study observed RCA occlusion in more than one-third of subjects which might correlate with the prevalence of POAF. We found left main stem involvement in 45% of the study population, which includes both significant (≥50% luminal stenosis) and non-significant (<50% luminal stenosis) left main disease. This relatively high prevalence of left main stem involvement most likely indicates the influence of the aging process on coronary circulation. The frequency of in-stent restenosis is 3%-20% of the study population [25]. Our observations match previous studies.

Coronary endarterectomy is associated with POAF [26]. One-third of the study population underwent coronary endarterectomy, which might influence the prevalence of POAF. We didn't find any obvious imbalance in mean serum electrolytes (Na+, K+, and Ca++) level up to the third postoperative day and assumed that electrolyte imbalance might not be associated with POAF. Athanasiou and colleagues reported the incidence of POAF is 22% in elderly people undergoing OPCAB [27]. Advanced age independently predicts the prevalence of POAF in patients undergoing CABG [8]. In our study, new onset POAF has been seen in 48.3% elderly. The higher frequency of POAF in this study was similar to the observed frequency (20%-40%) by Kouchoukos and colleagues [1]. This study's higher prevalence of POAF strongly predicts the influence of advanced age-related association with POAF. The episodes duration of the majority of POAF was within the first 24 hours following OPCAB. Most of the observed POAFs were paroxysmal AF. We used amiodarone as a rhythm control strategy for managing POAF. With conservative management using amiodarone, 79.31% of the POAF reverted spontaneously to normal sinus rhythm. The mean ICU stays and duration of hospital stays were 3.43±1.61 and 10.03±2.12 days, respectively, comparable to the study conducted by Mathew and colleagues [28]. In this study, only one patient developed a stroke postoperatively as a consequence of POAF. No mortality was observed in this study.

This present study has a few limitations, such as this is an observational study conducted in a single center for two years with a small sample size, which may not represent the elderly population of Bangladesh and across the world. Furthermore, the patients were operated on by different surgeons with different techniques and preferences, which were completely the choice and judgment of the individual surgeon. These might lead to a selection bias that cannot be excluded from the variables analyzed. Besides, the use of epiaortic ultrasound to identify sites for proximal anastomosis and the flow probes to check graft patency following distal anastomosis couldn't be performed due to the unavailability of resources in Bangladesh. In addition, this study observed the postoperative outcomes during the hospital stay period only. Although, our cross-sectional analysis has shown a high prevalence of POAF following OPCAB in the elderly. We believe that a prospective randomized control trial with a large elderly population in Bangladesh would strengthen the findings of this current study.

## Conclusions

Elderly patients with multiple comorbidities should promptly raise concerns regarding a POAF following off-pump coronary artery bypass graft surgery, and preoperative managing those factors will be important in reducing the risk of POAF.

## Appendices

Data availability statement

Datasets are available on reasonable request from the corresponding author.

Author contribution statement

Kumar A: Conceptualization; research protocol development; facing the institutional review board (IRB); data collection; data curation; formal analysis; investigation; methodology; resources; visualization; writing-original draft; writing-review & editing; and final approval of this document to be published.

Ranjan R: Conceptualization; methodology; visualization; supervision; writing-review & editing, and final approval of this document to be published.

Adhikary AB: Conceptualization; methodology; resources; supervision; validation, writing-review & editing; and final approval of this document to be published.

## References

[1] Kouchoukos NT, Blackstone EH, Hanley FL, Kirklin JK (2013). Cardiac rhythm disturbance. Kirklin/Barratt-Boyes Cardiac Surgery.

[2] Bai Y, Wang YL, Shantsila A, Lip GY (2017). The global burden of atrial fibrillation and stroke: A systematic review of the clinical epidemiology of atrial fibrillation in Asia. Chest.

[3] Jidéus L (2001). Atrial fibrillation after coronary artery bypass surgery : a study of causes and risk factors. Uppsala.

[4] Liu P, Wang F, Ren S (2014). A propensity score analysis on the effect of on-pump versus off-pump coronary artery bypass grafting for patients with coronary artery disease. J Thorac Dis.

[5] Siebert J, Rogowski J, Jagielak D, Anisimowicz L, Lango R, Narkiewicz M (2000). Atrial fibrillation after coronary artery bypass grafting without cardiopulmonary bypass. European Journal of Cardio-Thoracic Surgery.

[6] Dieberg G, Smart NA, King N (2016). On- vs. off-pump coronary artery bypass grafting: A systematic review and meta-analysis. Int J Cardiol.

[7] Fleg JL, Strait J (2012). Age-associated changes in cardiovascular structure and function: a fertile milieu for future disease. Heart Fail Rev.

[8] Thorén E, Hellgren L, Jidéus L, Ståhle E (2012). Prediction of postoperative atrial fibrillation in a large coronary artery bypass grafting cohort. Interact Cardiovasc Thorac Surg.

[9] Pilatis ND, Anyfantakis ZA, Spiliopoulos K (2013). The role of BNP and CRP in predicting the development of atrial fibrillation in patients undergoing isolated coronary artery bypass surgery. ISRN Cardiol.

[10] Filardo G, Ailawadi G, Pollock BD (2016). Sex differences in the epidemiology of new-onset in-hospital post-coronary artery bypass graft surgery atrial fibrillation: A large multicenter study. Circ Cardiovasc Qual Outcomes.

[11] Xu S, Zhang J, Xu YL, Wu HB, Xue XD, Wang HS (2017). Relationship between angiotensin converting enzyme, apelin, and new-onset atrial fibrillation after off-pump coronary artery bypass grafting. Biomed Res Int.

[12] Kinoshita T, Asai T, Suzuki T, Kambara A, Matsubayashi K (2012). Preoperative hemoglobin A1c predicts atrial fibrillation after off-pump coronary bypass surgery. Eur J Cardiothorac Surg.

[13] Newby DE, Grubb NR, Bradbury A (2014). Cardiovascular disease. Davidson’s Principle & Practice of Medicine.

[14] Hravnak M, Hoffman LA, Saul MI, Zullo TG, Whitman GR, Griffith BP (2002). Predictors and impact of atrial fibrillation after isolated coronary artery bypass grafting. Crit Care Med.

[15] Hogue CW Jr, Creswell LL, Gutterman DD, Fleisher LA (2005). Epidemiology, mechanisms, and risks: American College of Chest Physicians guidelines for the prevention and management of postoperative atrial fibrillation after cardiac surgery. Chest.

[16] Orimo H, Ito H, Suzuki T, Araki A, Hosoi T, Sawabe M (2006). Reviewing the definition of “elderly”. Geriatrics and Gerontology International.

[17] Turk T, Vural H, Eris C, Ata Y, Yavuz S (2007). Atrial fibrillation after off-pump coronary artery surgery: a prospective, matched study. J Int Med Res.

[18] Ahmad T, Alam MB, Khan A, Islam AM, Hossain Z, Asaduzzaman K (2017). Study on risk factors and pattern of coronary artery involvement in elderly acute coronary syndrome patients. Bangladesh Heart Journal.

[19] Akanda MA, Rahman S, Chowdhury AH, Zaman S, Ali MA, Sadequzzaman M (1996). Arteriographic pattern in coronary heart disease in Bangladesh demonstrated by selected coronary angiogram. Bangladesh Heart Journal.

[20] Banach M, Rysz J, Drozdz JA (2006). Risk factors of atrial fibrillation following coronary artery bypass grafting: a preliminary report. Circ J.

[21] Lewicki Ł, Siebert J, Rogowski J (2016). Atrial fibrillation following off-pump versus on-pump coronary artery bypass grafting: Incidence and risk factors. Cardiol J.

[22] Akazawa T, Nishihara H, Iwata H, Warabi K, Ohshima M, Inada E (2008). Preoperative plasma brain natriuretic peptide level is an independent predictor of postoperative atrial fibrillation following off-pump coronary artery bypass surgery. J Anesth.

[23] Tavakol M, Hassan KZ, Abdula RK (2009). Utility of brain natriuretic peptide as a predictor of atrial fibrillation after cardiac operations. Ann Thorac Surg.

[24] Caretta Q, Mercanti CA, De Nardo D, Chiarotti F, Scibilia G, Reale A, Marino B (1991). Ventricular conduction defects and atrial fibrillation after coronary artery bypass grafting. Multivariate analysis of preoperative, intraoperative and postoperative variables. European Heart Journal.

[25] Dangas GD, Claessen BE, Caixeta A, Sanidas EA, Mintz GS, Mehran R (2010). In-stent restenosis in the drug-eluting stent era. J Am Coll Cardiol.

[26] Güvenç O, Göncü MT, Engin M, Çayır MÇ, Özyazıcıoğlu AF (2019). Effects of coronary endarterectomy on postoperative early results in long segment coronary artery disease. The European Research Journal.

[27] Athanasiou T, Aziz O, Mangoush O (2004). Do off-pump techniques reduce the incidence of postoperative atrial fibrillation in elderly patients undergoing coronary artery bypass grafting?. Ann Thorac Surg.

[28] Mathew JP, Parks R, Savino JS, Friedman AS, Koch C, Mangano DT, Browner WS (1996). Atrial fibrillation following coronary artery bypass graft surgery: Predictors, outcomes, and resource utilization. JAMA.

